# Simultaneous ^18^F-labeled AlF-FAPI PET/MR images targeting the myocardial fibrosis in coronary artery disease and degenerative mitral valve regurgitant participants with left ventricular mechanical dyssynchrony

**DOI:** 10.1016/j.clinsp.2025.100624

**Published:** 2025-03-25

**Authors:** YuFeng Chen, Jia Guo, YuJi Zhang, DengShun Tao, KeYan Zhao, QingXue Shi, GuoXu Zhang, HuiShan Wang

**Affiliations:** a Department of Nuclear Medicine, General Hospital of Northern Theater Command, 83rd Wenhua Rd, Shenhe District, Shenyang, PR China; b Cardiovascular Surgery, General Hospital of Northern Theater Command, 83rd Wenhua Rd, Shenhe District, Shenyang, PR China

**Keywords:** ^18^F-AlF-FAPI, Fibrosis, LV mechanical dyssynchrony, PET/MR, Degenerative mitral valve regurgitation, Coronary artery disease

## Abstract

**Background:**

Myocardial fibrosis contributes to LV mechanical dyssynchrony and fibroblast activation protein is considered as a specific biomarker related to tissue remodeling. The authors aimed to explore the relationship between LVMD and myocardial fibrosis, and patterns of LV fibrosis distribution in CAD and Degenerative Mitral Valve Regurgitant (DMVR) participants by the technique of ^18^F-AlF-FAPI PET/MR.

**Methods:**

37 CAD and DMVR participants with LVMD and normal ones underwent cardiac PET/MR imaging, the correlations between SUV_mean_ of FAPI-uptakes and score/percentage of LV wall thickening, peak myocardial strains, and displacements were analyzed in PET/MR images, and ejection fractions and function parameters of mitral valve were compared.

**Results:**

There was inverse correlation between SUV_mean_ and score/percentage of LV wall thickening, peak circumferential and short-axial radial myocardial strains, and global peak myocardial strains and ejection fractions showed a decrease significantly in participants with heart diseases. In CAD participants, FAPI was distributed in the ischemic coronary arteries regions. Functions of myocardium in LAD and RCA regions were sensitive to fibrosis, however, those in LCX regions were resistant to fibrosis. In DMVR participants, high uptakes of FAPI were the locations of the valve annulus and papillary muscles, and SUV_mean_ in the basal inferospetal/basal inferior segments were a positive correlation with function parameters of the mitral valve.

**Conclusion:**

Uptake of ^18^F-AlF-FAPI in the myocardium could detect fibrosis and predict LVMD in CAD and DMVR participants. Moreover, there were different distributions of FAPI-uptake in those participants. In DMVR participants, SUV_mean_ in the basal inferospetal/basal inferior segments could assess dysfunction of the mitral valve.

## Highlights

• Uptake of ^18^F-FAPI in the myocardium can detect fibrosis and predict LV dyssynchrony.
• There are different distributions of FAPI-uptake in CAD and DMVR patients.
• FAPI-uptake in the anterior, septal and inferior walls can detect cardiac dysfunction.
• SUV_mean_ of FAPI in the lateral wall can determine the progression of cardiac dysfunction.
• FAPI-uptake in the basal inferospetal/basal inferior walls can assess the level of MR.

## Introduction

Ventricular dyssynchrony encompasses both electrical and mechanical dyssynchrony. Electrical dyssynchrony is characterized by a conduction delay resulting in a QRS duration exceeding 120 ms on the surface electrocardiogram. Mechanical dyssynchrony refers to the difference in the timing of mechanical contraction or relaxation within the Left Ventricle (LV) or between the LV and Right Ventricle (RV), and can result in suboptimal ventricular filling, a reduction in ventricular contractility, a greater degree and prolonged duration of mitral regurgitation, and paradoxical septal wall motion.^1^ Although electrical and mechanical dyssynchrony frequently coincide, Left Ventricular Mechanical Dyssynchrony (LVMD) is present in approximately 30%–40% of patients with normal QRS duration, with a greater frequency in patients with Coronary Artery Disease (CAD). Assessing Given that mechanical dyssynchrony is a marker of early myocardial damage and is independently associated with impaired cardiac function and poor prognosis in patients with Heart Failure (HF) and/or CAD, assessing mechanical dyssynchrony is crucial for selecting candidates for Cardiac Resynchronization Therapy (CRT).^2^

The pathological process of ventricular mechanical dyssynchrony may involve excessive myocardial fibrosis, eventually leading to myocardial dysfunction and even HF.^3^ To compensate for increasing pressure influenced by hemodynamic changes in the early stages, adaptive ventricular fibrosis is initiated to normalize wall stress, which preserves the wall structure and prevents fatal complications. However, at later stages, with the decompensation of ventricular function, excessive fibrosis can severely impair ventricular systolic function, further exacerbating ventricular mechanical dyssynchrony, ultimately leading to HF or sudden cardiac death. Currently, qualitative assessment of ventricular mechanical dyssynchrony involves echocardiographic evaluation, specifically through the identification of a septal flash on M-mode echocardiography or apical rocking on 2D echocardiography.^4^ The resolution of these findings is a prognostic marker, indicating a positive response to CRT. Quantitative assessment of ventricular mechanical dyssynchrony is even more complex. The simplest method involves measuring the septal-to-posterior wall motion delay using M-mode in the parasternal short-axis view. Several additional echocardiographic indices have been reported.^5^ A common method involves measuring the differences in the time-to-peak systolic velocity between opposing ventricular walls by assessing tissue velocity using tissue Doppler imaging or by evaluating myocardial strain using speckle-tracking echocardiography. However, these typical practice patterns vary widely between institutions.^6^ Obtaining accurate estimates of the overall prevalence based on assessment practices and interobserver variability is challenging. Although Cardiovascular Magnetic Resonance (CMR) imaging with myocardial tagging techniques can provide circumferential and longitudinal myocardial activation data along all three dimensions of the heart, and late gadolinium enhancement imaging can reveal the characterization and distribution of myocardial scars, gadolinium enhancement imaging fails to produce reproducible data on myocardial scars because it relies on subjective criteria rather than quantified ones.^7^

Although CMR imaging and echocardiography can be used to evaluate abnormal wall motion alterations, they cannot accurately reflect the underlying origins or mechanisms of ventricular mechanical dyssynchrony induced by fibrosis. Fibroblast Activation Protein (FAP) is a type II membrane-bound glycoprotein belonging to the dipeptidyl peptidase 4 family with both dipeptidyl peptidase and endopeptidase activities, which is considered a specific biomarker for tissue remodeling. When resting fibroblasts are activated by various pathological conditions, such as wound healing, they express high levels of FAP on the cellular membrane and secrete more extracellular matrix.^8^ Considering the promising roles of FAP in tissue remodeling, a small-molecule Fibroblast Activation Protein Inhibitor (FAPI), ^18^F-labeled AlF-FAPI, has been developed as a new-generation radiopharmaceutical. These are used for PET imaging to diagnose and treat tumors and non-malignant diseases associated with remodeling of the extracellular matrix.^8^

This study investigates the relationship between LVMD and myocardial fibrosis and the patterns of fibrosis distribution in LV in patients with CAD and Degenerative Mitral Valve Regurgitation (DMVR) with LVMD using Positron Emission tomography/Magnetic Resonance (PET/MR) with ^18^F-labeled AlF-FAPI.

## Methods

### Study participants

This prospective observational study included patients diagnosed with heart disease between August and December 2021. Based on the predetermined inclusion and exclusion criteria, the participants were divided into three groups: the DMVR group (patients with LVMD and DMVR), the CAD group (patients with LVMD and CAD), and the control group (healthy individuals without any heart disease).

### Inclusion criteria

Patients aged ≥ 18-years with evidence of left ventricular motion dyssynchrony on two-dimensional echocardiography and who had not undergone invasive interventions for heart disease (including percutaneous coronary intervention, catheter ablation, and coronary artery bypass grafting) were included. In addition, their blood pressure and lipid levels were consistently within the normal range regardless of medication use. Patients who met the following criteria were included in the DMVR group: 1) Two-dimensional echocardiography indicated primary mitral regurgitation due to degenerative changes in mitral valve structure (such as leaflet thickening, redundant prolapse, and calcification; 2) Doppler echocardiography detected a reflux bundle with a mitral regurgitant jet area > 4 cm^2^ and regurgitation fraction > 20%. Patients who met the following criteria were included in the CAD group: 1) Electrocardiography (ECG) revealed ST-segment changes (elevation or depression) and flattened or inverted T-waves; 2) Two-dimensional echocardiography indicated weakened left ventricular wall motion; and 3) Coronary Angiography (CAG) revealed > 50% stenosis in any coronary artery. If CAG results were unavailable, the criteria required that the degree of stenosis in any coronary artery indicated by Computed Tomography Coronary Angiography (CTCA) exceeded 50%, along with the presence of clinical manifestations related to myocardial ischemia (such as chest pain after exercise).

### Exclusion criteria

The exclusion criteria were as follows: age ˂ 18-years; secondary mitral regurgitation; patients with two or more valvular diseases, congenital heart disease, rheumatic heart disease, infectious endocarditis, cardiomyopathy, pericardial effusion, autoimmune diseases, and electrocardiographic evidence of bundle branch block; and inability to successfully complete CTCA, CAG, or PET/MR examinations. Additionally, patients with ST-segment changes (elevation or depression) on the ECG or abnormalities suggested by CTCA were excluded from the DMVR group. Patients with valvular disease were excluded from the CAD group.

**Fig. 1:**
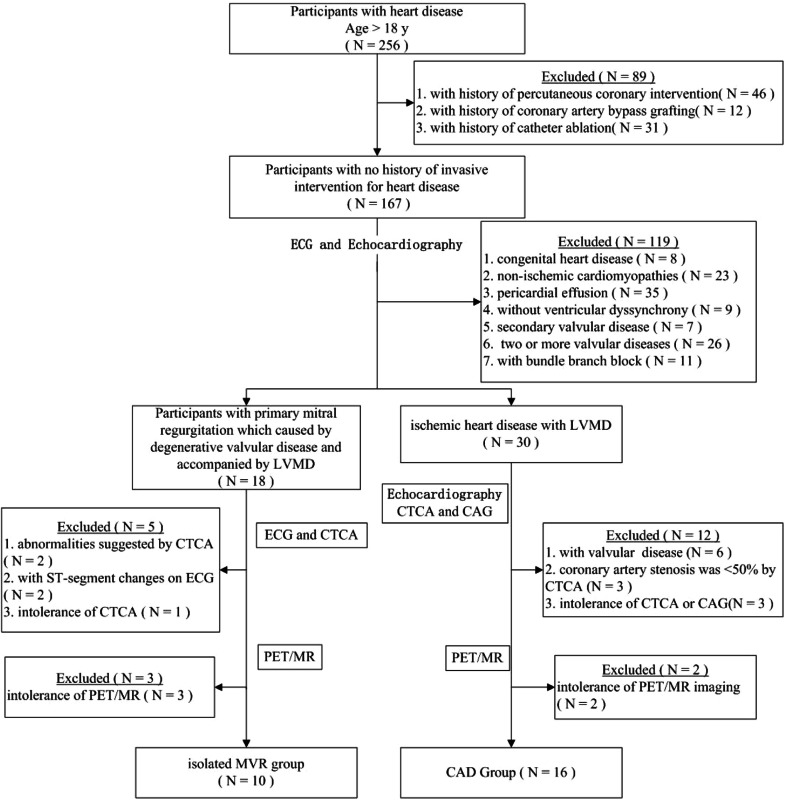
Participants selection flowchart. CAD, Coronary Artery Disease; CAG, Coronary Arteriography; CTCA, Computed Tomography Coronary Angiography; MVR, Mitral Valve Regurgitation; PET/MR, Positron Emission Tomography/Magnetic Resonance.

Additionally, age- and sex-matched individuals with no echocardiographic evidence of cardiovascular or other metabolic diseases and those who had not undergone invasive cardiac intervention were included as controls. All enrolled participants underwent cardiac ^18^F-AlF-FAPI PET/MR imaging (Fig. 1). This study was approved by the Ethics Committee of the General Hospital of Northern Theater Command (Y2021–012) and was conducted in accordance with the guidelines outlined in the Declaration of Helsinki. Written informed consent was obtained from all the participants.

### 18F-AlF-FAPI PET/MR imaging protocols

The precursor of ^18^F-AlF-FAPI (^18^F-AlF-NOTA-FAPI-42) with high chemical purity (> 95%) was provided by Jiangsu Huayi Technology Co., Ltd. (Jiangsu, China). Radiolabeling of ^18^F-AlF-FAPI was performed according to established protocols.^9^

Dynamic simultaneous cardiac PET/MRI was performed using an integrated 3.0-T MR scanner (Signa PET/MR, GE Healthcare, Waukesha, WI, USA). All participants received 2.5–3.0 MBq/kg ^18^F-AlF-FAPI intravenously, followed by PET/MR imaging at 40-min post-injection. The MR sequence parameters are as follows: 1) A three-dimensional, two-point Dixon, fast Spoiled Gradient Recalled echo (SPGR) sequence (LAVA‐Flex) was used for an MR attenuation correction (slice thickness 5.2 mm and gap 2.6 mm). 2) MR imaging encompassed long-axis (two- and four-chamber) and short-axis (covering the entire heart) views during end-expiratory breath holds with the following parameters: Field of View (FOV: 38 cm), Repetition Time (TR: 3.8 ms), minimum echo time, matrix size: 160 × 224, and slice thickness: 8 mm without interslice gaps. Two-dimensional ECG-gated steady‐state acquisition (FIESTA) was used for continuous short-axis coverage of the entire LV for co-registration and anatomical evaluation, with an acquisition of 30 frames per cardiac cycle. 3D ECG-gated PET data were reconstructed (voxel size: 2 × 2 × 2 mm) using a Time-Of-Flight, Point Spread Function, Ordered Subset Expectation Maximization (TOF-PSF-OSEM) algorithm with a matrix of 192 × 192, iterations 3, subsets 28, a Gaussian filter width of 8 mm, and 8 frames per cardiac cycle. The PET and MR scans were performed simultaneously under cardiac gating, which minimized the motion effect. In addition, the PSF model was used in PET reconstruction to reduce partial volume effects.^10^ Two nuclear physicians independently assessed ^18^F-FAPI PET/MR images, and disagreements, if any, were resolved by consensus.

### 18F- AlF-FAPI PET/MR image interpretation and analysis

#### PET data analysis

ECG-gated PET data were analyzed using CardIQ Physio (GE AW4.7, USA), which autodetects valve planes and myocardial borders, allowing for manual refinement. According to the American Heart Association standard ,^11^ the LV myocardial wall was segmented into 17 sectors in short-axis cardiac PET polar maps. For participants in the control and CAD groups, these segments were classified according to the coronary artery supply: Left Anterior Descending Artery (LAD), Right Coronary Artery (RCA), and Left Circumflex Artery (LCX).^12^ In the DMVR group, the segments were split into two groups based on their attachment to the mitral annulus and papillary muscles. SUV_mean_, a semiquantitative index, was calculated for each myocardial segment by averaging the Standardized Uptake Values (SUVs) across the layers in the transverse fused PET/MR images. In patients with CAD or DMVR, the 17 segments were divided into three or two groups, and FAPI uptake was represented by the regional mean SUV_mean_. Wall thickening was semi-quantitatively analyzed using percentage ranges and converted into scores. Because the guidelines of the American Heart Association have emphasized that there is tremendous variability in the coronary artery blood supply to myocardial segments, and the greatest variability occurs at the apex, which can be supplied by any of the three arteries, the data from the 17th myocardial segment were excluded in the polar map from the short-axis view of cardiac PET images.^11^ (Details are provided in the Supplementary Material).

#### CMR data analysis

Following automatic delineation of the endocardium and epicardium on the short- and long-axis cardiac MR images using uAI Discover Cardiac MR software (Version: R001; Shanghai United Imaging Healthcare Co., Ltd.) and subsequent verification by two experienced imaging physicians, the software automatically calculated the percentage of systolic LV wall thickening, peak myocardial strains (peak circumferential, longitudinal, short-axial radial and long-axial radial strains), corresponding displacements in each segment, and the global peak myocardial strains (the global peak circumferential, longitudinal, short-axial radial and long-axial radial strains).^13^

#### Correlative analysis of FAPI-uptake and MVR parameters

According to the 2017 ESC/EACTS guidelines and the 2017 ACC expert consensus decision pathway on the management of MVR, regurgitant volume, regurgitant fraction, and regurgitant orifice area were used to assess the impact of FAPI-uptakes on the function of the mitral valve. Standard echocardiography and CMR were used to measure the regurgitant fraction, regurgitant orifice area, regurgitant volume, and ejection fraction, respectively. Correlations between the SUV_mean_ in the most susceptible myocardial segments were analyzed using Spearman correlation analysis.

### Statistical analysis

All statistical analyses were performed using SPSS 22.0 software (IBM Corp., Armonk, NY, USA). Spearman correlation analysis was performed to analyze the correlation between SUV_mean_ and the score of systolic LV wall thickening on PET images of all participants. Additionally, correlations between SUV_mean_ and percentage of systolic LV wall thickening, four peak myocardial strains, and the corresponding displacements of each myocardial segment in the MR images were separately analyzed using Spearman correlation analysis. Fisher's precision probability test was used to compare the global peak myocardial strains and Left Ventricular Ejection Fraction (LVEF) between patients with heart disease and healthy individuals, and to compare differences in the four peak myocardial strains in the basal inferoseptal, basal inferior, mid inferior, and mid anterolateral segments between patients with DMVR and healthy individuals. To compare FAPI-uptake SUV_mean_ among participants, the continuity variable was assessed for normality using the Shapiro-Wilk test, and Levene's test was used for equality of variance. Pairwise comparisons were conducted using either Mann-Whitney *U* tests or independent samples *t*-tests. Results are presented as means ± SDs, and a two-tailed p-value ˂0.05 indicated statistical significance.

## Results

### Study cohort

Among the 256 patients with heart diseases, 89 who had undergone invasive interventions were excluded (percutaneous coronary intervention [*n* = 46], coronary artery bypass grafting [*n* = 12], and radiofrequency ablation [*n* = 31]). The remaining 167 patients, without a history of invasive intervention for heart disease, were screened using echocardiography. Based on the echocardiogram results, 52 patients with the following conditions were excluded (congenital heart disease [*n* = 8], cardiomyopathy [*n* = 23], pericardial effusion [*n* = 35], and combination of valvular and ischemic heart disease [*n* = 41]). The remaining participants were divided into two groups: patients with valvular disease and LVMD (*n* = 39) and patients with ischemic heart disease and LVMD (*n* = 21). Furthermore, after excluding 26 patients with two or more valvular diseases in the valvular disease group, the remaining 13 patients were included in the isolated DMVR group. Of the 21 patients in the ischemic heart disease group, 18 were included in the pure CAD group after exclusion of three patients who were intolerant to CTA examination. Moreover, three and two patients from the isolated DMVR and pure CAD groups, respectively, were excluded owing to intolerance to CMR examination. Finally, 10 patients with DMVR were included in the group of isolated DMVR (mean age, 57 ± 15 years). The CAD group included 16 patients (mean age, 62 ± 10 years). Additionally, 11 age- and sex-matched healthy volunteers (mean age, 54 ± 7 years) were included as controls. The detailed characteristics of the participants are summarized in Table 1 and Fig. 1.

**Table 1:** Demographics characteristics of study participants at evaluation.

	Participants with CAD (*n* = 16)	Participants with DMVR (*n* = 10)	Normal Participants (*n* = 11)
**Age (y)**	61.56 ± 10.45	56.78 ± 15.42	54.40 ± 6.59
**Sex**			
Male	10 (62.5)	7 (70)	5 (45.45)
Female	6 (37.5)	3 (30)	6 (54.55)
**BMI (kg/m^2^)**	28.54±5.59	22.75±2.37	23.45±2.09
**Clinical characteristics**			
*Symptoms*			
Chest pain	16 (100)	4 (40)	0 (0)
Palpitations	16 (100)	5 (50)	0 (0)
*Comorbidities*			
Hypertension	14 (87.5)	4 (40)	0 (0)
Smoker	9 (56.25)	4 (40)	0 (0)
Hyperlipidemia	10 (62.5)	4 (40)	0 (0)
Congestive heart failure	4 (25)	3 (30)	0 (0)
Diabetes	2 (12.5)	1 (10)	0 (0)
Atrial fibrillation	2 (12.5)	6 (60)	0 (0)
Creatinine (μmoL/L)	81.7 ± 12.7	87.1 ± 14.5	83.4 ± 11.4
**NYHA functional class**			
1	2 (12.5)	1 (10)	0 (0)
2	9 (56.25)	6 (60)	0 (0)
3	3 (18.75)	3 (30)	0 (0)
4	2 (12.5)	0 (0)	0 (0)
**Medical therapy**			
ACE inhibitor	5 (31.25)	4 (40)	0 (0)
β-Blockers	6 (37.5)	6 (60)	0 (0)
Oral anticoagulants	3 (18.75)	2 (20)	0 (0)
Aspirin	4 (25)	2 (20)	0 (0)
Nitrates	14 (87.5)	2 (20)	0 (0)
Statins	9 (56.25)	5 (50)	0 (0)
**Echocardiography**			
LVEDV (mL)	109.31±38.04	145.30±34.74	114.61±23.07
LVESV (mL)	62.88±20.07	78.80±15.80	41.78±10.67
SV (mL)	46.44±18.32	66.50±21.47	72.82±22.86
LVEF (%)	41.85±3.27	44.83±5.79	62.47±10.94
CO (L/min)	3.21±1.12	4.61±1.37	4.96±1.47
Mitral leaflet flail	0 (0)	6 (60)	0 (0)
Mitral valve prolapsed	0 (0)	8 (80)	0 (0)
Regurgitant Volume (mL/beat)	12.71±4.03	47.85±18.06	10.91±4.74
Regurgitant Jet Area (cm^2^)	3.06±0.21	6.75±1.45	2.01±0.17
Regurgitant fraction (%)	10.63±2.94	35.22±10.31	9.23±2.01
**CTCA characteristics**			
1 vessel ≥ 50%	7 (43.75)	0 (0)	0 (0)
2 vessel ≥ 50%	3 (18.75)	0 (0)	0 (0)
3 vessel ≥ 50%	2 (12.5)	0 (0)	0 (0)
Presence of vascular stenosis and all less than 50%	4 (25)	0 (0)	0 (0)
**CAG characteristics**			
1 vessel ≥ 50%	8 (50)	NA	NA
2 vessel ≥ 50%	5 (31.25)	NA	NA
3 vessel ≥ 50%	3 (18.75)	NA	NA

Values are n (%) or mean ± SD.

ACE, Angiotensin-Converting Enzyme; CAD, Coronary Artery Disease; CAG, Coronary Arteriography; CTCA, Computed Tomography Coronary Angiography; LVEDV, Left Ventricular End-Diastolic Volume; LVESV, Left Ventricular End-Systolic Volume; DMVR, Degenerative Mitral Valve Regurgitation; NA, No Data; NYHA, New York Heart Association; SV, Stroke Volume; LVEF, Left Ventricular Ejection Fraction; CO, Cardiac Output.

### Relationship between SUV_mean_ of FAPI uptake and LVMD in patients with heart disease and healthy controls

**Fig. 2:**
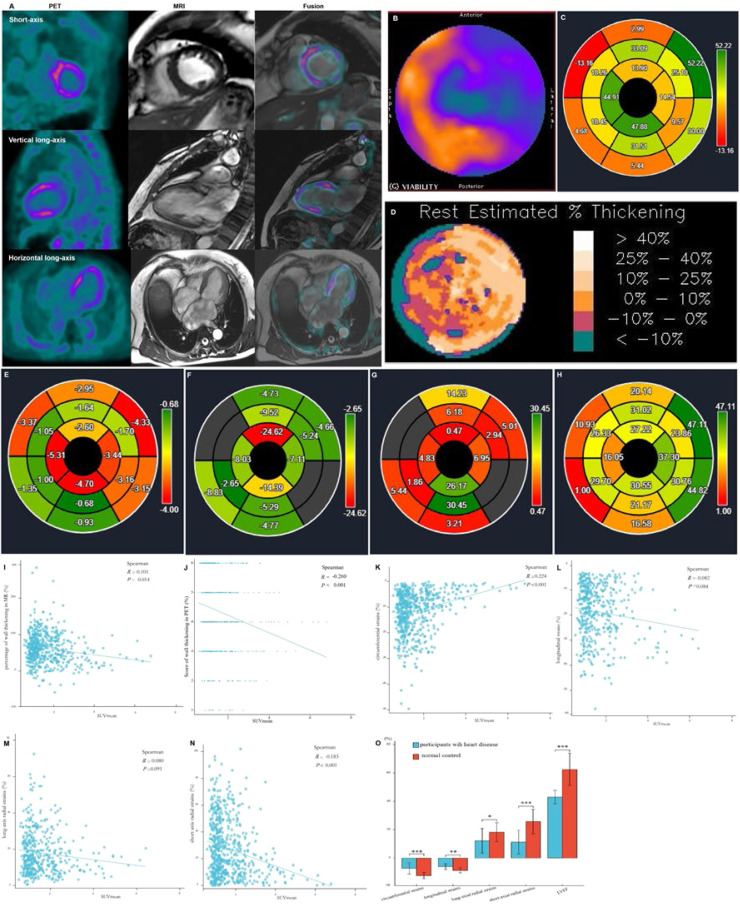
Images in a 64-year-old woman with multiple coronary stenosis (A‒H). ^18^F-AlF-FAPI PET images, MR images, and PET/MR fusion images in short-axis, vertical long-axis, and horizontal long-axis cine (A). The distribution of FAPI uptake in the polar map of PET images (B). There were higher FAPI uptake in the septum and inferior myocardium. The polar map of the percentages of systolic LV wall thickening per myocardial segment in MR images (C). The polar map of the percentage ranges of systolic LV wall thickening in PET images (D). Both C and D showed that the wall thickening decreased in the septum and inferior myocardium. The polar maps of the peak circumferential, longitudinal, long-axis radial and short-axis radial strains per myocardial segment in MR images (E‒H). The correlation analysis between the percentages and scores of systolic LV wall thickening, the peak circumferential, longitudinal, long-axis radial and short-axis radial strains and SUV_mean_ in 16 myocardial segments from all participants (I‒N). There were significant negative correlation between SUV_mean_ and the percentages (I) and scores (J) of systolic LV wall thickening, the peak circumferential (K) and short-axial radial strains (N), respectively. The comparison of the global circumferential, longitudinal, long-axis radial and short-axis radial strains and the Left Ventricular Ejection Fraction (LVEF) between all participants with heart diseases and normal ones (O). Each column represents mean ± SD. Asterisks indicate significant differences between them. * *p* < 0.05, ** *p* < 0.01, *** *p* < 0.001.

Except for the apical myocardium segment, data obtained from 592 myocardial segments in the polar map from the short-axis view of the cardiac PET/MR images of all participants were analyzed using statistical methods. Data on the FAPI distribution in the LV wall, percentage range of systolic wall thickening, percentage of systolic LV wall thickening, and peak myocardial strains in the 592 myocardial segments were visualized in the corresponding polar maps generated from the short-axis view of cardiac PET and MR images using the software (Fig. 2A‒H). A significant negative correlation was observed between SUV_mean_ and score of systolic LV wall thickening in PET images (*r* = -0.260, *p* < 0.001) and SUV_mean_ and percentage of systolic LV wall thickening in MR images (*r* = -0.101, *p* = 0.014). These findings indicate that increased FAPI uptake in the cardiac tissues was associated with decreased systolic LV wall thickening in the corresponding tissues, thereby reducing the magnitude of myocardial deformation of LV wall motion (Fig. 2, Fig. 2).

Furthermore, analysis of short- and long-axis CMR images demonstrated an inverse correlation between FAPI uptake in each segment and the peak circumferential and short-axial radial strains (SUV_mean_ and circumferential strain: *r* = 0.224, *p* < 0.001; SUV_mean_ and short-axis radial strain: *r* = -0.183, *p* < 0.001). With an increase in FAPI uptake in the cardiac tissues, the peak circumferential and short-axial radial strains in the corresponding tissues decreased. However, there were no significant differences in the correlations between the SUV_mean_ and the peak longitudinal and long-axial radial strains in the same FAPI uptake segment (SUV_mean_ and longitudinal strain: *r* = -0.082, *p* = 0.084; SUV_mean_ and long-axis radial strain: *r* = -0.080, *p* = 0.091) (Fig. 2K‒N).

Comparative analysis of the global peak circumferential, longitudinal, long-axis radial and short-axis radial strains and LVEF between patients with heart disease and healthy controls revealed significant differences (circumferential strain: -7.38 ± 3.99 vs. -12.43 ± 2.14 [*p* < 0.001], longitudinal strain: -6.12 ± 1.99 vs. -8.82 ± 1.70 [*p* = 0.0014], long-axis radial strain: 12.14 ± 8.62 vs. 18.18 ± 6.66 [*p* = 0.0183], short-axis radial strain: 11.22 ± 8.40 vs. 25.70 ± 8.66 [*p* < 0.001], LVEF: 43.00 ± 4.74 vs. 62.47 ± 11.47 [*p* < 0.001]). Specifically, all global peak strains and LVEF were significantly lower in patients with heart disease than in healthy controls (Fig. 2O).

### Distributions of FAPI uptake in patients with CAD

**Fig. 3:**
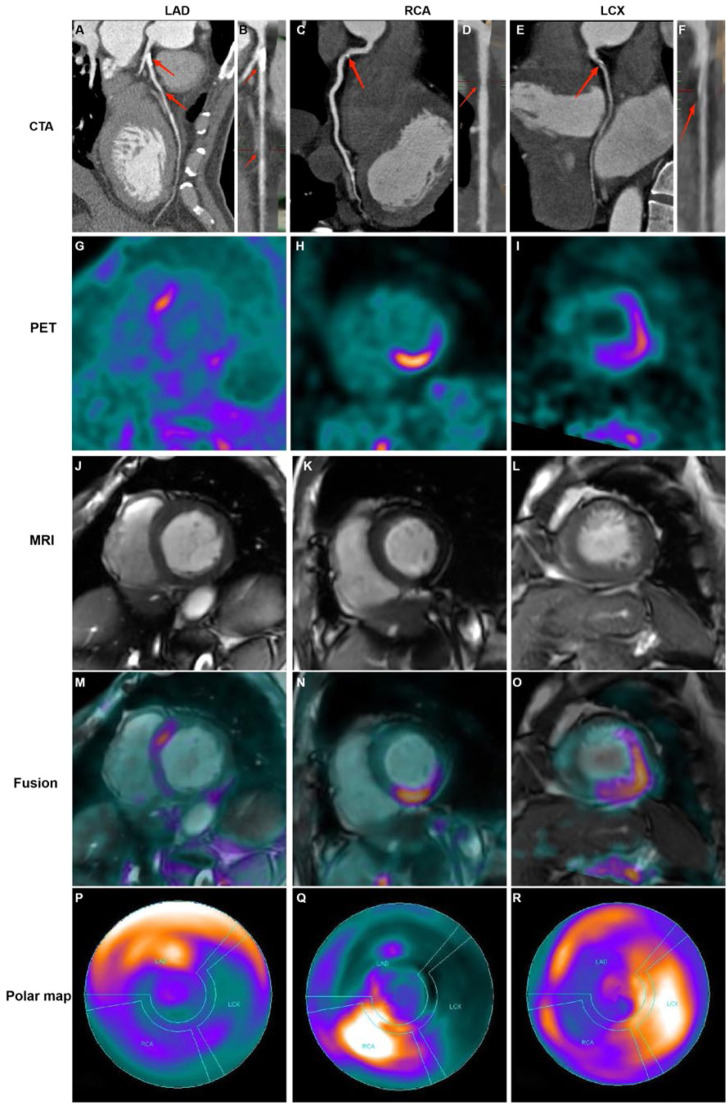
Representative images in participants with coronary stenosis. Curved reconstruction map (A, C, E) and probe map (B, D, F) obtained from Computed Tomography Angiography (CTA) showed the vascular stenosis (indicated by red arrow). ^18^F-FAPI PET images (G, H, I), MRI images (J, K, L), and PET/MR fusion image (M, N, O) in short-axis cine showed that FAPI was up-taken in a certain slicer. The distribution of FAPI-uptake in the left ventricle was presented with a polar map (P, Q, R). The first column showed images in a 42-year-old man with 50% stenosis in Left Anterior Descending coronary artery (LAD) and 30% stenosis in Right Coronary Artery (RCA). In the short-axis cardiac PET/MR fusion images, the uptake of FAPI in the septum was higher (M), and in the polar map of PET images, the anterior and septal wall had higher uptake (P). The second column showed images in a 61-year-old woman with 40% stenosis in RCA. FAPI-uptake increased in the inferoseptal and inferior wall in the short-axis cardiac PET/MR fusion images and the polar map of PET images (N and Q). The third column showed images in a 67-year-old man with 50% stenosis in Left Circumflex Artery (LCX), 30% stenosis in LAD and 30% stenosis in RCA. The FAPI uptake was significantly increase in the lateral wall, and slightly increase in the anterior, septal and inferior wall in the short-axis cardiac PET/MR fusion images and the polar map of PET images (O and R).

**Fig. 4:**
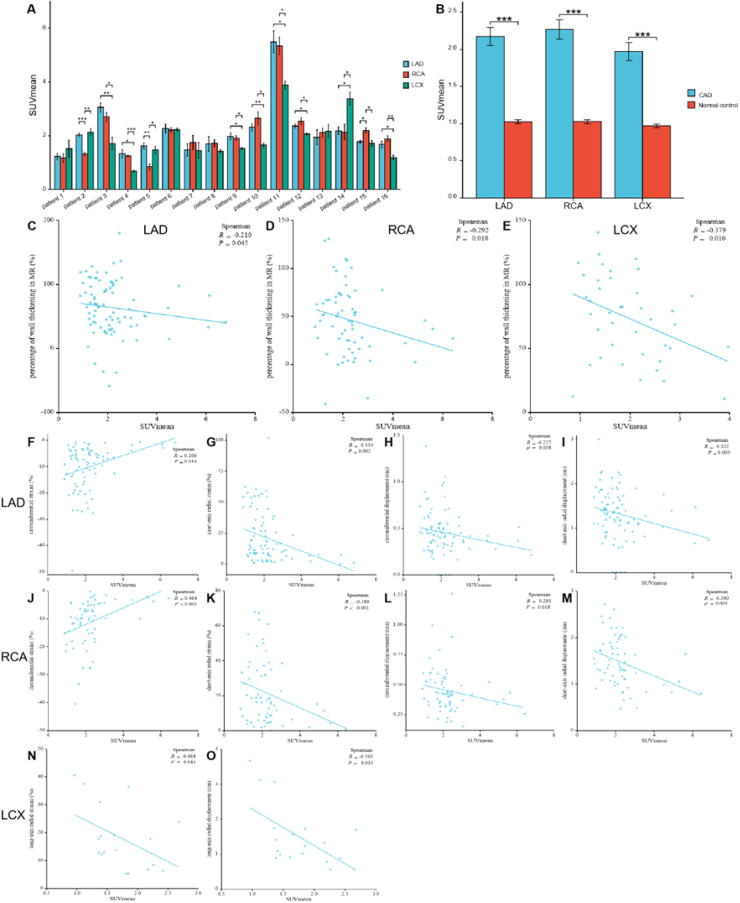
The comparison of SUV_mean_ in the myocardium fed by every coronary artery in each participant with Coronary Artery Disease (CAD) (A) For each participant, FAPI uptake was significantly higher in the myocardium fed by the narrow coronary artery than that fed by normal coronary artery. The comparison of SUV_mean_ between in the myocardium fed by the coronary artery with stenosis in CAD participants and in those fed by the corresponding normal coronary artery in normal ones (B). It showed that uptake of FAPI in the myocardium fed by every coronary artery with stenosis was significantly higher than that in the same myocardium fed by non-occluded coronary artery. The correlation analysis between percentage of wall thickening and SUV_mean_ in the myocardium fed by anterior descending coronary artery (LAD), Right Coronary Artery (RCA) and Left Circumflex Artery (LCX), respectively (C‒E). The correlation analysis between the peak circumferential and short-axis radial strains and displacements and SUV_mean_ in LAD and RCA, respectively (F‒M). The correlation analysis between the long-axis radial strains and displacements and SUV_mean_ in LCX (N‒O). Each column represents mean ± SD. Asterisks indicate significant differences between them. * *p* < 0.05, ** *p* < 0.01, *** *p* < 0.001.

The LV myocardial wall was divided into three parts based on the blood-supplying areas of the main coronary arteries, LAD, RCA, and LCX areas, which are shown in the polar maps from the short-axis view of the cardiac PET images (Fig. 3). Myocardial regions supplied by coronary arteries diagnosed as stenotic by CTA exhibited abnormally high FAPI uptake. Moreover, FAPI-uptake was significantly higher in areas supplied by the occluded coronary artery than in those with normal blood supply in the same CAD patients (all p-values ˂ 0.05; Fig. 4A and Table 2).

**Table 2:** The characteristics of participants’ sex, age, examinations of CTCA and SUV_mean_ of ^18^F-FAPI PET images in 16 participants with coronary artery disease.

Patient	Sex	Age (y)	CTCA			CAG			SUV_mean_^a^		
			LAD (%)	RCA (%)	LCX (%)	LAD (%)	RCA (%)	LCX (%)	Myocardium fed by LAD	Myocardium fed by RCA	Myocardium fed by LCX
Patient 1	M	51	95	50	80	99	50	100	1.23 ± 0.28	1.17 ± 0.36	1.52 ± 0.71
Patient 2	M	67	50	0	30	80	0	40	2.04 ± 0.14	1.32 ± 0.14	2.13 ± 0.30
Patient 3	M	42	100	60	0	100	100	60	3.05 ± 0.42	2.70 ± 0.38	1.71 ± 0.50
Patient 4	M	67	70	60	0	90	60	0	1.34 ± 0.36	1.25 ± 0.07	0.68 ± 0.08
Patient 5	M	78	70	0	30	80	0	40	1.62 ± 0.26	0.84 ± 0.23	1.47 ± 0.28
Patient 6	F	64	40	40	30	60	40	40	2.27 ± 0.41	2.21 ± 0.21	2.22 ± 0.12
Patient 7	M	65	70	60	80	95	60	90	1.47 ± 0.54	1.75 ± 0.59	1.45 ± 0.66
Patient 8	F	65	80	30	40	90	40	40	1.70 ± 0.67	1.72 ± 0.30	1.43 ± 0.13
Patient 9	F	56	80	100	0	90	100	0	1.97 ± 0.31	1.92 ± 0.18	1.53 ± 0.07
Patient 10	F	66	75	30	0	80	50	0	2.32 ± 0.32	2.65 ± 0.57	1.65 ± 0.16
Patient 11	M	42	50	30	0	50	30	0	5.49 ± 0.99	5.43 ± 0.73	3.89 ± 0.34
Patient 12	F	79	40	30	0	60	40	0	2.36 ± 0.18	2.54 ± 0.28	2.06 ± 0.11
Patient 13	M	67	30	30	50	40	60	60	1.94 ± 0.67	2.12 ± 0.36	2.17 ± 0.58
Patient 14	M	54	0	0	40	0	0	60	2.19 ± 0.34	2.14 ± 0.65	3.37 ± 0.54
Patient 15	F	61	0	40	0	0	60	0	1.77 ± 0.15	2.20 ± 0.21	1.72 ± 0.24
Patient 16	M	72	60	40	0	100	95	0	1.67 ± 0.30	1.89 ± 0.23	1.19 ± 0.19

a Data are means ± SDs. %, The extent of stenosis. M, Male; F, Female; CAG, Coronary Arteriography; CTCA, Computed Tomography Coronary Angiography; LAD, Left Anterior Descending coronary artery; LCX, Left Circumflex Artery; RCA, Right Coronary Artery; SUV_mean_, Mean Standardized Uptake Values.

Comparison between CAD patients and healthy controls revealed significantly higher FAPI uptake in ischemic myocardial regions supplied by stenotic coronary arteries than those fed by non-occluded vessels. The SUV_mean_ from the myocardium fed by the same coronary artery in the CAD and healthy controls were as follows: LAD: 2.16 ± 1.12 vs. 1.02 ± 0.21; RCA: 2.27 ± 1.06 vs. 1.03 ± 0.19; LCX: 1.97 ± 0.76 vs. 0.97 ± 0.18 (all *p* < 0.01; Fig. 4B).

Analysis of corresponding abnormal blood-supplying myocardial areas delineated by the three major coronary artery branches in the polar maps from the short-axis view of the CMR images revealed inverse correlations between the percentage of systolic LV wall thickening and FAPI uptake (SUV_mean_) (LAD, *r* = -0.210, *p* = 0.045; RCA, *r* = -0.292, *p* = 0.018; LCX, *r* = -0.379, *p* = 0.016) (Fig. 4C‒E). Moreover, the peak circumferential and short-axis radial strains and displacement of the corresponding orientation in the LAD- and RCA-supplied myocardial areas were inversely correlated with FAPI uptake (SUV_mean_). The results were as follows: peak circumferential and short-axis radial strains for LAD, *r* = 0.266, *p* = 0.014 and *r* = -0.334, *p* = 0.002; for RCA, *r* = 0.464, *p* < 0.001 and *r* = -0.289, *p* < 0.001, respectively; and circumferential and short-axis radial displacements for LAD, *r* = -0.227, *p* = 0.038 and *r* = -0.322, *p* = 0.003; for RCA, *r* = -0.293, *p* = 0.018 and *r* = -0.390, *p* = 0.001 (Fig. 4F‒M). In addition, the peak long-axis radial strain and displacement in the LCX-supplied myocardial areas were inversely correlated with FAPI uptake (SUV_mean_). The results were as follows: *r* = -0.464, *p* = 0.043 and *r* = -0.565, *p* = 0.015, respectively (Fig. 4N‒O).

### Distributions of FAPI uptake in patients with DMVR

**Fig. 5:**
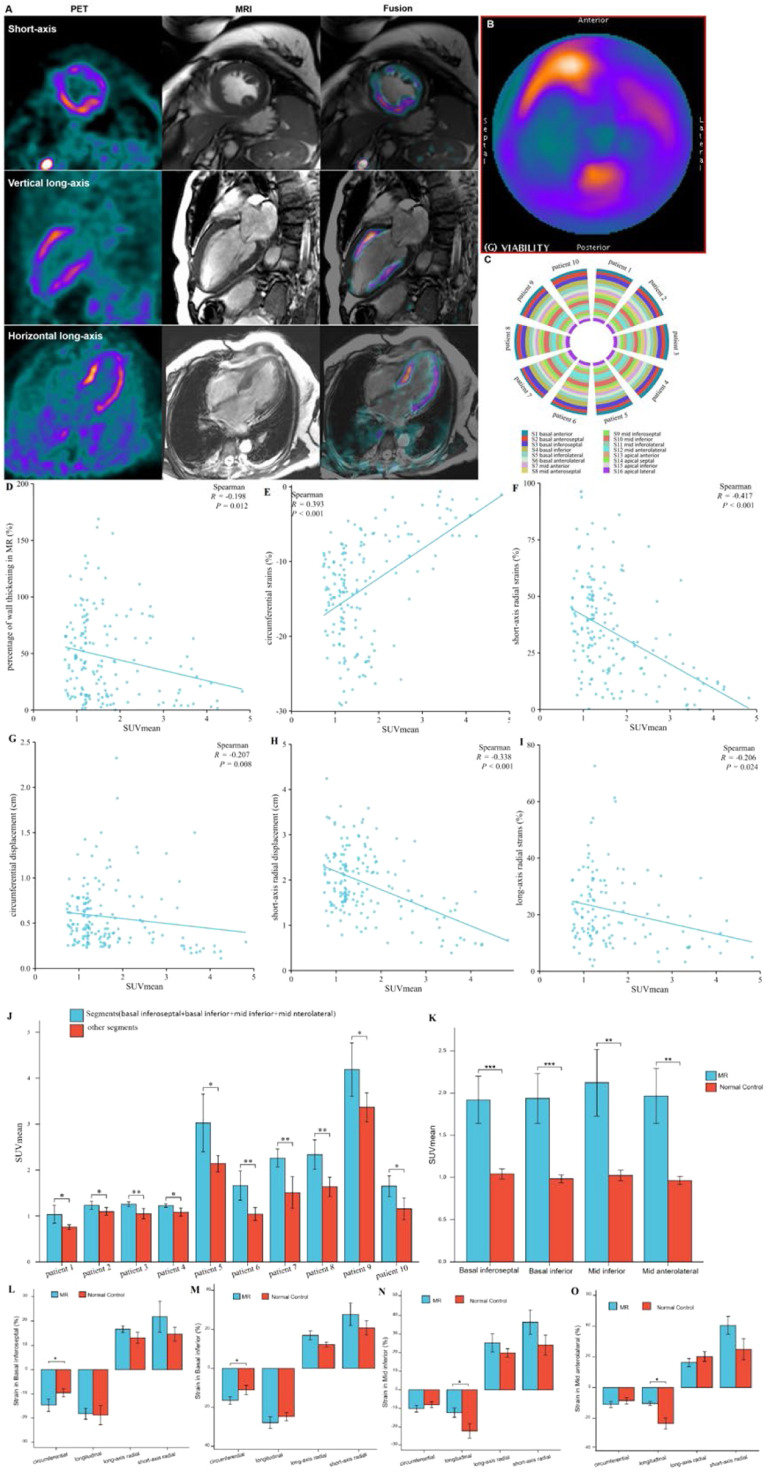
Images in a 63-year-old man with Mitral Valve Regurgitation (MVR) (A and B). ^18^F-AlF-FAPI PET images, MR images, and PET/MR fusion images in short-axis, vertical long-axis, and horizontal long-axis cine (A). The distribution of FAPI uptake in the polar map of PET images (B). There were higher FAPI uptakes in the middle myocardium, especially in the mid anterior, mid inferior and mid anterolateral wall. The uptakes of FAPI in each myocardial segment in the polar map of PET images from all participants with MVR (C). The value of SUV_mean_ of FAPI-uptakes in each segment was represented by the width of each color bar. It showed that for each participant with MVR, there were no significant differences in FAPI-uptakes between each myocardial segment. The comparison of SUV_mean_ between two parts of all myocardial segments in each participant with MVR (D). In each participant, SUV_mean_ of FAPI uptakes in the part of myocardial segments, including basal inferoseptal, basal inferior, mid inferior and mid anterolateral wall, was higher than that in the other part including the remaining myocardial segments. The comparison of SUV_mean_ in the basal inferoseptal, basal inferior, mid inferior and mid anterolateral wall between all participants with MR and normal ones (E). There was significant differences in SUVmean of each myocardial segments between them. The correlation analysis between percentage of wall thickening and SUV_mean_ in the all myocardial segments from MVR participants (F). The correlation analysis between the peak circumferential, short and long-axis radial strains and displacements and SUV_mean_ in the all myocardial segments from MVR participants (G‒K). The comparison of the peak circumferential, longitudinal, long-axis radial and short-axis radial strains in the basal inferoseptal (L), basal inferior (M), mid inferior (N) and mid anterolateral wall (O) between all participants with MR and normal ones. Each column represents mean ± SD. Asterisks indicate significant differences between them. *p < 0.05, **p < 0.01, ***p < 0.001.

The results from the polar map of cardiac PET images from the patients with DMVR indicated that the distribution of FAPI uptake was multifocal and irregular in the myocardial segments, with no significant differences in FAPI uptake (SUV_mean_) between individual myocardial segments (Fig. 5A‒C). However, for each DMVR patient, the sum of the SUV_mean_ of FAPI uptake within segments attached to the annulus of the mitral valve and papillary muscles (basal inferoseptal, basal inferior, mid inferior, and mid anterolateral walls) was significantly higher than that in the other myocardial segments (p ˂ 0.05) (Fig. 5D). Moreover, comparison between patients with DMVR and controls revealed significantly higher FAPI-uptake (SUV_mean_) in the basal inferoseptal, basal inferior, mid inferior, and mid anterolateral wall segments of individuals with DMVR (1.92 ± 0.28 vs. 1.04 ± 0.06 in the basal inferoseptal wall, 1.94 ± 0.30 vs. 0.98 ± 0.05 in the basal inferior wall, 2.12 ± 0.39 vs. 1.02 ± 0.21 in the mid inferior wall, and 1.97 ± 0.33 vs. 0.96±0.05 in the mid anterolateral wall; all *p* < 0.01; Fig. 5E).

Analysis of short-axis CMR polar maps revealed a significant negative correlation between the percentage of systolic LV wall thickening and FAPI uptake (SUV_mean_) in all myocardial segments of DMVR patients (*r* = -0.198, *p* = 0.012; Fig. 5F). Moreover, the peak circumferential and short-axis radial strains and displacement of the corresponding orientation in all myocardial segments were inversely correlated with FAPI (SUV_mean_). The results were as follows: peak circumferential and short-axis radial strains, *r* = 0.393, *p* < 0.001 and *r* = -0.417, *p* < 0.001, and circumferential and short-axis radial displacement, *r* = -0.207, *p* = 0.008 and *r* = -0.338, *p* < 0.001 (Fig. 5G‒J). Although the peak long-axis radial strains in all myocardial segments were inversely correlated with the SUV_mean_ of FAPI (*r* = -0.206, *p* = 0.024), no significant correlation was observed between the long-axis radial displacement and SUV_mean_ of FAPI (Fig. 5K).

In addition, data from the basal inferoseptal, basal inferior, mid inferior and mid anterolateral walls from the short-axis view of CMR images revealed significant differences in the peak circumferential strains in both the basal inferoseptal and basal inferior walls between patients with DMVR and healthy controls (-14.74 ± 2.55 vs. -9.58 ± 1.66 and -16.51 ± 1.96 vs. -11.11 ± 2.35; all *p* < 0.05). Comparison between DMVR patients and controls revealed significantly lower peak longitudinal strains in both the mid inferior (-12.24 ± 2.50 vs. -22.10 ± 3.92) and mid anterolateral wall segments of DMVR patients (-10.33 ± 1.36 vs. -23.30 ± 3.59; *p* < 0.05; Fig. 5L‒O).

### Correlation of SUV_mean_ in the myocardial segments of high FAPI uptake with MVR parameters

**Fig. 6:**
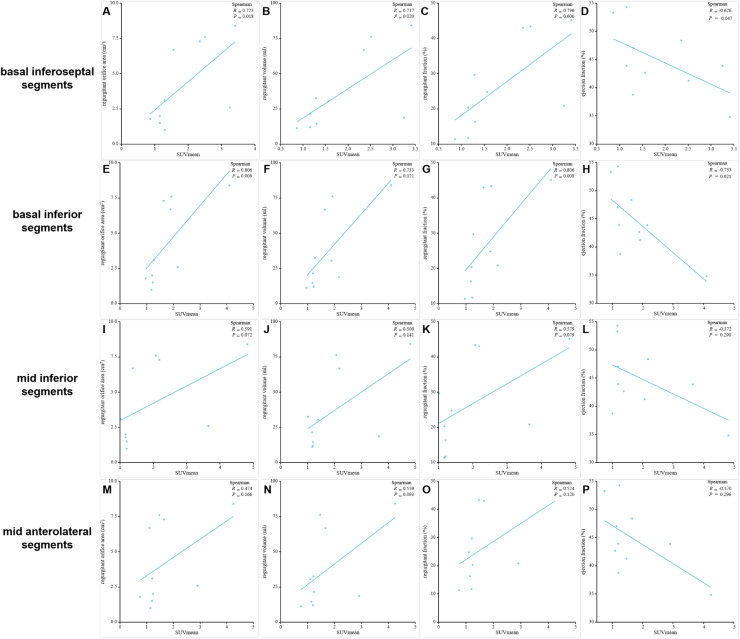
The correlation analysis between regurgitant volume, regurgitant fraction, regurgitant orifice area, ejection fraction and SUV_mean_ in the basal inferoseptal (A‒D), basal inferior (E‒H), mid inferior (I‒L) and mid anterolateral segments (M‒P) from MVR participants.

Positive correlations were observed between SUV_mean_ of FAPI and regurgitant volume, regurgitant fraction, and regurgitant orifice area in the basal inferoseptal and basal inferior segments. Conversely, a negative correlation was observed between SUV_mean_ of FAPI and ejection fraction within these segments. These results were as follows: *r* = 0.723, *p* = 0.018 and *r* = 0.806, *p* = 0.008 for regurgitant orifice area in the basal inferoseptal and basal inferior segments; *r* = 0.717, *p* = 0.020 and *r* = 0.733, *p* = 0.021 for regurgitant volume in the basal inferoseptal and basal inferior segments; *r* = 0.796, *p* = 0.006 and *r* = 0.806, *p* = 0.008 for regurgitant fraction in the basal inferoseptal and basal inferior segments; and *r* = -0.626, *p* = 0.047, and *r* = -0.733, *p* = 0.021 for ejection fraction in the basal inferoseptal and basal inferior segments (Fig. 6A‒H and Table 3). However, the SUV_mean_ of FAPI in the mid-inferior and mid-anterolateral segments was not significantly correlated with these parameters of mitral regurgitation (all *p* < 0.05; Fig. 6I‒P).

**Table 3:** The Characteristics of participants’ sex, age and examinations of CMR in 10 participants with DMVR.

Patient	Sex	Age (y)	LVSV (mL/beat)	RVSV (mL/beat)	EF (%)	Regurgitant Volume (mL/beat)	Regurgitant orifice área (cm^2^)	Regurgitant fraction (%)
Patient 1	M	20	98.9	87.63	53.29	11.27	1.8	11.40
Patient 2	M	66	102.99	90.92	43.93	12.07	1.5	11.72
Patient 3	F	68	48.3	57.07	38.75	32.59	3.1	29.72
Patient 4	M	64	146.02	45.37	54.30	21.56	2	20.34
Patient 5	M	66	179.82	61.08	43.89	18.74	2.6	20.86
Patient 6	M	59	149.24	74.66	47.06	14.58	1	16.34
Patient 7	M	68	118.73	99.46	41.24	76.27	7.6	43.40
Patient 8	F	57	145.14	54.36	48.37	66.78	7.3	43.04
Patient 9	F	46	52.02	52.34	34.82	84.3	8.4	45.17
Patient 10	M	63	92.21	116.64	42.67	30.43	6.7	24.81

M, Male; F, Female; LVSV, Left Ventricular Stroke Volume; RVSV, Right Ventricular Stroke Volume; EF, Ejection Fraction.

Regurgitant Volume (mL/beat) = LVSV (mL/beat) - RVSV (mL/beat).

Regurgitant fraction (%) = Regurgitant Volume (mL/beat) / LVSV (mL/beat) × 100%.

## Discussion

Considering the advantages of radionuclide imaging for quantitative analyses of the tracer concentration, higher temporal resolution, greater reproducibility, and volumetric assessment of both ventricles, Single-Photon Emission Computed Tomography (SPECT) myocardial perfusion scintigraphy in combination with measures of both perfusion and function has been recently used to evaluate ventricular synchrony and predict CRT outcomes.^14^^,^^15^ Chen et al. adapted ^99m^Tc-tetrofosmin scintigraphy to demonstrate the different dyssynchrony patterns in normal, ischemic, and infarcted myocardium patients in response to pharmacological stress. In patients with ischemia, the parameters of ventricular dyssynchrony in the stress scan were significantly larger than in the rest scan.^16^ Moreover, compared with pre-therapy scans, post-therapy 99mTc-MIBI perfusion scans have demonstrated improvements in ventricular dyssynchrony among patients with chronic HF and bradycardia.

Current research on ventricular mechanical dyssynchrony using radionuclide imaging technology mainly focuses on evaluating the effect of hemodynamics on ventricular mechanical dyssynchrony after coronary artery ischemia.^15^ However, in addition to coronary artery disease, many patients with valvular heart disease and dilated and restrictive cardiomyopathy exhibit signs of ventricular mechanical dyssynchrony accompanied by different levels of myocardial fibrosis in the ventricular wall. Because of the lack of specific radiopharmaceuticals, comprehensive and reproducible studies evaluating the beneficial effects of fibrosis on ventricular mechanical dyssynchrony are limited. With the development of small-molecule FAP inhibitors such as ^18^F-labeled AlF-FAPI, research on the relationship between the abnormality of ventricular wall motion and the alteration of myocardial fibrosis has become feasible using PET imaging technology. Therefore, this study aimed to elucidate the association between LVMD and myocardial fibrosis and explore different FAPI distribution patterns in the LV of CAD and DMVR patients with LVMD using ^18^F-labeled AlF-FAPI PET/MR imaging.

### Outcomes of PET/MR

FAP, a specific biomarker of activated fibroblasts, can accurately predict the process of fibrosis in injured myocardial tissues. In this study, the activated fibroblast cells in the LV wall were clearly visualized using ^18^F-labeled AlF-FAPI in the PET/MR images, and the SUV_mean_ of FAPI in the images semi-quantitatively indicated the degrees of FAP expression in the activated fibroblast cells. In the polar maps from the short-axis view of cardiac PET images, the score represented by the percentage range of systolic wall thickening in the high SUV_mean_ segments showed a significant difference compared to that in the low SUV_mean_ segments. The score and SUV_mean_ demonstrated a negative correlation, indicating that the extent of myocardial fibrosis is higher in low-score segments. The accumulation of activated fibroblasts, which transdifferentiate into myofibroblasts contributes to fibrosis that reduces ventricular compliance and increases ventricular stress, ultimately leading to diastolic and systolic dysfunction.^6^ Although wall stress is a major determinant of systolic myocardial deformation and ventricular ejection, it is not directly measurable and must be inferred. Several assumptions for wall stress have been proposed, which are more complex than the others, but most of them are based on measurable parameters such as wall thickness. During the systolic dysfunction, the regional end-systolic wall stress was negatively correlated with systolic wall thickening.^17^^,^^18^ These PET image results were consistent with those of the MR images. In the polar maps from the short-axis views of the CMR images, the percentages of systolic LV wall thickening were significantly lower in the high-uptake FAPI region than in the low/null-uptake FAPI region, which could be measured by myocardial deformation of the LV wall motion.

Systolic myocardial strains are invariably diminished in the region of myocardial fibrosis, with reduced longitudinal and circumferential shortening and diminished/absent systolic wall thickening.^17^ These results from the short-axis and long-axis views of CMR images also showed that the levels of short-axis radial and circumferential strains in the regions of high-uptake FAPI decreased more than those in the regions of low/null-uptake FAPI. In contrast, there was a shift towards higher regional stresses evaluated by the myocardial deformation of myocardial contractility in the region of high-uptake FAPI. Interestingly, the results from the longitudinal strains in the regions of high-uptake FAPI on MRI were inconsistent with those in the literature ,^17^ which could be attributed to the compensation of some myocardial segments. Moreover, the alteration of strains in each segment of the LV wall enhanced by high uptake of FAPI could produce abnormal global strains in the ventricular wall, which could contribute to LVMD and lead to an abnormal ventricular ejection fraction.

### Distributions of FAPI abnormality and plausible mechanisms in CAD

In the CAD group, polar maps from the short-axis view of cardiac PET images revealed a predominant distribution of high FAPI uptake within myocardial segments supplied by the main coronary artery afflicted with ischemia. Cardiomyocyte survival and function are susceptible to changes in blood flow. In patients with CAD, owing to myocardial ischemia, cardiomyocytes in areas of abnormal coronary arteries are more susceptible to dysfunction and even death. A large number of injured cardiomyocytes can stimulate inflammation and the subsequent activation of fibroblasts, leading to scar formation. Necrotic cardiomyocytes were replaced by collagen-based scars, causing fibrosis.^6^^,^^19^^,^^20^ Moreover, in ischemic regions, cardiac tissue enhanced by FAPI uptake exhibited inhomogeneous alterations in functional parameters. Specifically, the circumferential and short-axis radial strains and displacements decreased in the septal, anterior, and inferior segments, and long-axis radial strains and displacements decreased in the lateral segments, which could contribute to LVMD. The most susceptible fibrosis-producing segment could not be observed in each independent ischemic region, indicating that global remodeling and extensive fibrosis were induced in each ischemic region. Interestingly, long-axis radial strains and displacements, other than circumferential ones, decreased in the LCX-supplied myocardial areas during ischemia, suggesting that the myocardium in the lateral segments may be more resistant to the influence of fibrosis than that in the anterior, septal, and inferior segments, as circumferential shortening is the first principal strain at the end of systole.^17^ This further indicates that the lateral myocardial segments may play a compensatory role in preserving normal cardiac function when the function of other segments is abnormal in the context of fibrosis. In contrast, the anterior, septal, and inferior myocardial segments are more susceptible to fibrosis, particularly ischemia. Therefore, the extent of myocardial fibrosis in the lateral myocardial segments might effectively predict the progression of cardiac dysfunction; however, the extent of myocardial fibrosis in the anterior, septal, and inferior myocardial segments may be more sensitive in detecting abnormalities in cardiac function.

### Distributions of FAPI abnormality and plausible mechanisms in DMVR

In the DMVR group, the distribution of FAPI uptake showed a distinct pattern. The results from the polar maps of the short-axis view of cardiac PET images indicated that although segments of FAPI-uptakes showed multifocal and irregular, with common high uptake areas located at the valve annulus and papillary muscles (basal segments and midwall segments), indicating that MVR induces focal LV remodeling and fibrosis.

In addition, changes in the percentages of systolic LV wall thickening, circumferential and short-axis radial strains, and displacements of the corresponding orientation were negatively correlated with FAPI uptake. Variations in these strains were observed at the valve annulus and papillary muscles. The altered organization of the extracellular matrix is the common structural hallmark of degenerative mitral regurgitation. Degenerative mitral regurgitation can be classified into fibroelastic deficiency and myxomatous degeneration.^21^ Concurrent with leaflet alterations, patients with degenerative mitral regurgitation often exhibit severe mitral valve annular abnormalities, including annular dilatation and disjunction. These anatomical annular alterations lead to abnormal annular displacement, characterized by outward movement during late systole, termed as functional prolapse. Additionally, annular flattening increases the stress applied to the leaflets and chordae tendineae.^22^ Normally, the motion of the mitral annulus is passive and determined by the contraction and relaxation of the adjacent atrial and ventricular musculature. Consequently, under normal conditions, the posterior mitral ring and its adjacent myocardium move downward and anteriorly during systole, synchronizing with the remainder of the LV. In contrast, patients with mitral valve prolapse exhibit a peculiar functional abnormality of the mitral annulus (i.e., unusual systolic curling of the posterior mitral ring on the adjacent myocardium). The systolic movement of the ring was primarily downward with little, if any, anterior motion, resulting in a curled appearance in real-time motion, which may increase the circumferential strains of the mitral ring and adjacent myocardium. The presence of a systolic curling motion may contribute to the paradoxical increase in the mitral valve annulus diameter during systole, myxomatous degeneration of the leaflets, and myocardial stretch in the LV inferobasal wall and papillary muscles with relative hypertrophy and fibrosis.^23^ The increased longitudinal distance between the mitral valve and papillary muscles in degenerative mitral regurgitation decreases the longitudinal myocardial strains of the papillary muscles and its adjacent myocardium in systole. Chronic abnormal hemodynamic alteration induced by mitral regurgitation aggravates LV cardiac tissue remodeling and increases interstitial fibrosis, increasing the susceptibility of fibrosis in the papillary muscles and valve annulus.^22^ These factors directly contributed to the development of LVMD.

The European and American guidelines for the definition of severe mitral regurgitation use regurgitant orifice area, regurgitant volume, and regurgitant fraction for quantification and risk stratification.^24^^,^^25^ However, their clinical utility is limited.^26^ Given the intrinsic relationship between the basal inferoseptal, basal inferior, mid inferior and mid anterolateral myocardial segments and the anatomic locations of papillary muscles and valve annulus and their increased susceptibility to fibrosis in mitral regurgitation, the correlations between these parameters and SUV_mean_ of FAPI in these myocardial segments were analyzed. Notably, FAPI uptake and regurgitant volume, regurgitant fraction, regurgitant orifice area and ejection fraction demonstrated strong correlations in the basal inferoseptal and basal inferior segments These findings indicate that myocardial fibrosis in these two segments could directly impact mitral valve function. Notably, FAPI uptake in the basal inferoseptal and basal inferior segments serves as a potential surrogate marker for additional risk stratification and regurgitation quantification in degenerative mitral regurgitation.

### Study limitations

This study had several limitations. First, the relatively small sample size might have contributed to observation bias. Second, the quantitative relationship between the SUV_mean_ of FAPI uptake in the PET images and the extent of fibrosis in the cardiac tissues could not be determined, which merits further evaluation using PET/MRI combined with immunohistochemistry and western blotting. Third, this study did not include patients with different levels of CAD and DMVR; therefore, the clinical implications of the FAPI uptake in the basal inferoseptal and basal inferior segments in patients with DMVR and the extent of fibrosis in the anterior, septal, inferior, and lateral segments in patients with CAD could not be validated.

## Conclusions

The uptake of ^18^F-labeled AlF-FAPI in injured myocardial tissues accurately detected fibrosis in CAD and DMVR patients with LVMD. Moreover, there were different patterns of FAPI uptake distribution in the LV of patients with DMVR and CAD. The basal inferoseptal, basal inferior, mid inferior, and mid anterolateral segments were more susceptible to fibrosis in patients with DMVR, and SUV_mean_ in the basal inferoseptal and basal inferior segments accurately assessed mitral valve dysfunction.

## Data availability

The datasets generated during the current study are available from the corresponding authors on reasonable request.

## Ethics approval

This study was performed in line with the principles of the Declaration of Helsinki and the CONSORT Statement rules, and was approved by the ethics committee of the General Hospital of Northern Theater Command (Y2021–012).

## Consent to participate

Informed consent was obtained from all individual participants included in the study.

## Consent to publish

The authors affirm that human research participants provided informed consent for the publication of the images in Figs. 2, Fig. 3, Fig. 5.

## Funding

This work was supported by the Plan of Liaoning Province Applied and Basic Scientific Research (grant number 2022JH2/101500011) and the Shenyang Science and Technology Plan Project (22–321–33–33).

## CRediT authorship contribution statement

**YuFeng Chen:** Conceptualization, Writing – review & editing. **Jia Guo:** Data curation. **YuJi Zhang:** Formal analysis. **DengShun Tao:** Project administration. **KeYan Zhao:** Resources. **QingXue Shi:** Methodology. **GuoXu Zhang:** Funding acquisition. **HuiShan Wang:** Conceptualization, Supervision.

## Declaration of competing interest

The authors declare no conflicts of interest.

## References

[1] Lindenfeld J., Zile MR., Libby P. (2022). Braunwald's Heart Disease: A Textbook of Cardiovascular Medicine.

[2] Dalgaard F., Fudim M., Borges-Neto S., Di Carli M.F. (2021). Nuclear Cardiology and Multimodal Cardiovascular Imaging: A Companion to Braunwald's Heart Disease.

[3] Di Bella G., Siciliano V., Aquaro G.D., Molinaro S., Lombardi M., Carerj S. (2013). Scar extent, left ventricular end-diastolic volume, and wall motion abnormalities identify high-risk patients with previous myocardial infarction: a multiparametric approach for prognostic stratification. Eur Heart J.

[4] Dikdan S.J., Co M.L., Pavri BB. (2022). Dyssynchronous heart failure: a clinical review. Curr Cardiol Rep.

[5] Mele D., Luisi G.A., Malagù M., Laterza A., Ferrari R., Bertini M. (2018). Echocardiographic evaluation of cardiac dyssynchrony: does it still matter?. Echocardiography.

[6] MacDonald M.R., Hawkins N.M., Balmain S., Dalzell J., McMurrayJJV Petrie MC (2008). Transthoracic echocardiography: a survey of current practice in the UK. QJM.

[7] Haaf P., Garg P., Messroghli D.R., Broadbent D.A., Greenwood J.P., Plein S. (2016). Cardiac T1 mapping and extracellular volume (ECV) in clinical practice: a comprehensive review. J Cardiovasc Magn Reson.

[8] Dendl K., Koerber S.A., Kratochwil C., Cardinale J., Finck R., Dabir M. (2021). FAP and FAPI-PET/CT in malignant and non-malignant diseases: a perfect symbiosis?. Cancer.

[9] Wang S., Zhou X., Xu X., Ding J., Liu S., Hou X. (2021). Clinical translational evaluation of Al18FNOTA-FAPI for fibroblast activation protein-targeted tumour imaging. Eur J Nucl Med Mol Imaging.

[10] Petibon Y., Guehl N.J., Reese T.G., Ebrahimi B., Normandin M.D., Shoup T.M. (2017). Impact of motion and partial volume effects correction on PET myocardial perfusion imaging using simultaneous PET-MR. Phys Med Biol.

[11] Cerqueira M.D., Weissman N.J., Dilsizian V., Jacobs A.K., Kaul S., Laskey W.K. (2002). Standardized myocardial segmentation and nomenclature for tomographic imaging of the heart. A statement for healthcare professionals from the Cardiac Imaging Committee of the Council on Clinical Cardiology of the American Heart Association. Circulation..

[12] Grupper A., Gewirtz H., Kushwaha SS., Dilsizian V. (2017). Atlas of Cardiac Innervation.

[13] Yang D., Huang Q., Mikael K., Al’ Aref S., Axel L., Metaxas D. (2020). 2020 IEEE 17th International Symposium on Biomedical Imaging.

[14] Wassenaar R., O'Connor D., Dej B., Ruddy T.D., Birnie D. (2009). Optimization and validation of radionuclide angiography phase analysis parameters for quantification of mechanical dyssynchrony. J Nucl Cardiol.

[15] Naya M., Manabe O., Koyanagawa K., Tamaki N. (2018). The role of nuclear medicine in assessments of cardiac dyssynchrony. J Nucl Cardiol.

[16] Chen J., Garcia E.V., Folks R.D., Cooke C.D., Faber T.L., Tauxe E.L. (2005). Onset of left ventricular mechanical contraction as determined by phase analysis of ECG-gated myocardial perfusion SPECT imaging: development of a diagnostic tool for assessment of cardiac mechanical dyssynchrony. J Nucl Cardiol.

[17] Bogaert J., Bogaert J. (2012). Clinical Cardiac MRI.

[18] Bogaert J., Taylor AM., Bogaert J. (2012). Clinical Cardiac MRI.

[19] Woodall M.C., Woodall B.P., Gao E., Yuan A., Koch WJ. (2016). Cardiac fibroblast GRK2 deletion enhances contractility and remodeling following ischemia/reperfusion injury. Circ Res.

[20] Severino P., D'Amato A., Pucci M., Infusino F., Birtolo L.I., Mariani M.V. (2020). Ischemic heart disease and heart failure: role of coronary ion channels. Int J Mol Sci.

[21] Van Wijngaarden A.L., Kruithof B.P.T., Vinella T., Barge-Schaapveld DQCM, Ajmone Marsan N. (2021). Characterization of degenerative mitral valve disease: differences between fibroelastic deficiency and Barlow's disease. J Cardiovasc Dev Dis.

[22] Delgado V., Ajmone Marsan N., Bonow R.O., Hahn R.T., Norris R.A., Zühlke L. (2023). Degenerative mitral regurgitation. Nat Rev Dis Primers.

[23] Basso C., Perazzolo Marra M. (2018). Mitral annulus disjunction: emerging role of myocardial me-chanical stretch in arrhythmogenesis. J Am Coll Cardiol.

[24] Baumgartner H., Falk V., Bax J.J., De Bonis M., Hamm C., Holm P.J. (2017). 2017 ESC/EACTS guidelines for the management of valvular heart disease. Eur Heart J.

[25] Bonow R.O., O'Gara P.T., Adams D.H., Badhwar V., Bavaria J.E., Elmariah S. (2020). Focused update of the 2017 ACC expert consensus decision pathway on the management of mitral regurgitation. J Am Coll Cardiol.

[26] Lopes B.B.C., Kwon D.H., Shah D.J., Lesser J.R., Bapat V., Sarano M.E. (2021). Importance of myocardial fibrosis in functional mitral regurgitation: from outcomes to decision-making. JACC Cardiovasc Imaging.

